# Synergistic effects of high early-life stress exposure and HIV infection on reaction time variability

**DOI:** 10.3389/fpsyg.2023.1096266

**Published:** 2023-04-17

**Authors:** Jordan P. Sergio, Retina Kundu, Roger C. McIntosh, Mabel Palmero, Rachal R. Hegde, Marcel A. de Dios, Uraina S. Clark

**Affiliations:** ^1^Department of Neurology, Icahn School of Medicine at Mount Sinai, New York, NY, United States; ^2^Fordham University, New York, NY, United States; ^3^Department of Psychology, University of Miami, Miami, FL, United States; ^4^Department of Psychological, Health, and Learning Sciences, University of Houston, Houston, TX, United States

**Keywords:** adverse childhood experiences, childhood trauma, early life stress, reaction time variability, HIV

## Abstract

Addressing comorbidities contributing to cognitive impairment in people living with HIV (PLWH) remains imperative. Prior studies utilizing reaction time intra-individual variability (RT-IIV), a robust behavioral marker of cognitive dysfunction, demonstrate increased cognitive impairment in adults living with HIV who have high early life stress (ELS) exposure relative to those with low-ELS exposure. Yet, it is unknown whether RT-IIV elevations are due to high-ELS alone or both HIV-status and high-ELS. In the current study, we explore the potential additive effects of HIV and high-ELS exposure on RT-IIV to better characterize the independent and combined effects of these factors on RT-IIV among PLWH. We assessed 59 PLWH and 69 HIV-negative healthy control (HC) participants with either low or high ELS on RT-IIV during a working memory task (1-back). We observed a significant interaction between HIV status and ELS exposure on RT-IIV, PLWH who had experienced high ELS demonstrating RT-IIV elevations relative to all other groups. In addition, RT-IIV was significantly associated with ELS exposure in PLWH, but not in the HC group. We also observed associations between RT-IIV and measures of HIV-disease severity (plasma HIV viral load, nadir CD4) among PLWH. Taken as a whole, these findings provide novel evidence of the combined effects of HIV and high-ELS exposure on RT-IIV, and thus suggest HIV-related and ELS-related neural abnormalities may act in an additive or synergistic manner to affect cognition. Such data warrant further investigation into the neurobiological mechanisms associated with HIV and high-ELS exposure that contribute to increased neurocognitive dysfunction among PLWH.

## Introduction

The advancement of combination antiretroviral therapies (cART) has contributed to a decline in HIV-associated neurocognitive disorders (HAND). Yet, up to 50% of HIV infected individuals still experience varying degrees of cognitive impairment (Heaton et al., 2010). Understanding the role of comorbid factors in the etiology of cognitive impairment among people living with HIV (PLWH) is becoming increasingly important in the cART era with mounting evidence that comorbidities are associated with higher rates of cognitive impairment and contribute variability in the emergence and severity of dysfunction (McIntosh et al., 2023).

Several factors are known to contribute to the risk of cognitive impairment in adults living with HIV, including diabetes, hepatitis C virus (HCV) coinfection, and alcohol/substance misuse (e.g., Heaton et al., 2010). Emerging data indicate that psychosocial risk factors, such as early-life stress (ELS), also contribute to cognitive impairment among PLWH (Clark et al., 2012, 2017, 2023; Spies et al., 2012, 2016, 2017). High ELS exposure is associated with cognitive impairment among non-HIV samples (Philip et al., 2013). Such data suggest that examining ELS-related cognitive impairment in the context of HIV is particularly critical given evidence that PLWH are more likely to experience ELS relative to those without HIV (Spies et al., 2016; Clark et al., 2023). For example, it has been reported that, among men in the United States, each additional ELS event increased the odds of HIV infection by 1.32 times (Reisner et al., 2011).

With respect to the cognitive effects of ELS among PLWH, a recent study using reaction time intra-individual variability (RT-IIV) to assess cognition revealed greater cognitive impairment in PLWH with high-ELS exposure relative to those with low-ELS exposure (Clark et al., 2018). Further, this study found that cognitive impairment, as assessed by RT-IIV, correlated with lower whole-brain volumes among PLWH, aligning with prior data revealing RT-IIV as a useful indicator of neural integrity (MacDonald et al., 2006; Walhovd and Fjell, 2007; Jackson et al., 2012; Nilsson et al., 2014) and dysfunction (e.g., mild cognitive impairment; Collins and Long, 1996; MacDonald et al., 2006; Dixon et al., 2007; Duchek et al., 2009).

Reaction time intra-individual variability is a measure of an individual’s response-time variability across trials in a particular task. It is a sensitive and robust behavioral marker of cognitive dysfunction both in PLWH (Ettenhofer et al., 2010) and in non-HIV samples (Haynes et al., 2017). Elevated RT-IIV is thought to reflect attentional lapses, cognitive inefficiency, and cognitive instability (Ode et al., 2011; Tamm et al., 2012). Several studies have documented RT-IIV elevations among PLWH (Levine et al., 2006, 2008; Ettenhofer et al., 2010), and while ELS-related effects on RT-IIV have been reported in PLWH (Clark et al., 2018), none have investigated the potential additive or synergistic effects of HIV and high ELS on RT-IIV. Hence, it remains unclear whether the observed elevations in RT-IIV reflect ELS-related changes alone or the additive effects of HIV and high-ELS exposure on cognition. Clarifying this issue will help to provide greater understanding of the role high-ELS exposure plays in the etiology of cognitive impairment among PLWH as well as further characterize the potential utility of RT-IIV as a behavioral marker of cognitive function in PLWH, with respect to its sensitivity to both psychosocial (ELS) and biological (HIV) risk factors known to impact brain and cognition. Based on prior findings (e.g., Ettenhofer et al., 2010; Clark et al., 2012), we hypothesized that we would observe combined effects of HIV and high-ELS exposure on RT-IIV, such that PLWH with high ELS would exhibit greater RT-IIV elevations relative to all other groups (i.e., PLWH with Low-ELS, HIV-negative High-ELS, and HIV-negative Low-ELS groups).

## Methods

### Participants

The study included 59 HIV-seropositive (HIV) and 69 HIV-seronegative healthy control (HC) participants. HIV serostatus was documented by ELISA and confirmed by Western blot test. Participants were recruited from Icahn School of Medicine at Mount Sinai in New York, NY, United States and The Miriam Hospital in Providence, RI, United States. PLWH were recruited from affiliated clinics and local community groups; HC were recruited from community (e.g., local ads, word of mouth). Inclusion criteria were: age between 21 and 70 years, right-handed, completed ≥8 years of education, native English speaker. All participants obtained a score of ≥24 points on the Mini-Mental State Exam (MMSE). To avoid confounds that could potentially impact performance on cognitive tests, we excluded participation on the basis of reported history of developmental or learning disability; uncorrected abnormal vision; major psychiatric illness (e.g., schizophrenia, bipolar disorder); neurological illness affecting the central nervous system (e.g., stroke, progressive multifocal leukoencephalopathy); and traumatic head injury with a loss of consciousness of >10 min. Substance use exclusion criteria included reported current alcohol dependence; use of heroin/opiates or any intravenous drug within the past 6 months; use of cocaine within the past month; and positive urine toxicology at the time of assessment (cocaine, opiates, methamphetamine, amphetamine, benzodiazepine, barbiturates, methadone, and oxycodone). Individuals with substance use history were excluded as described above to enhance internal validity. One investigator (USC) oversaw all procedures. The research was approved by the Institutional Review Boards at the Icahn School of Medicine at Mount Sinai and The Miriam Hospital. All participants gave their informed written consent and received $125 compensation for their time upon completion of the study.

### Demographic measures

All participants provided their alcohol and drug use histories, which were quantified using the Kreek-McHugh-Schluger-Kellogg scale (KMSK; Kellogg et al., 2003). There were three subscales characterizing lifetime consumption of alcohol (KMSK-A), cocaine (KMSK-C), and opiates (KMSK-O). The Wechsler Test of Adult Reading (WTAR) estimated premorbid intellectual function (Wechsler, 2001); scaled scores were derived using published normative data. WTAR data were unavailable for four participants (one HC, three HIV). Participants were assessed for HCV infection, defined as positive HCV antibody. HIV-disease characteristics collected from PLWH participants included HIV disease duration, nadir CD4 levels (i.e., the lowest ever CD4 T-cell count), and antiretroviral (ARV) use via self-report and verified against the medical record. Current CD4 levels and plasma HIV viral loads (HIVL) were obtained from medical records. HIVL was log 10 transformed to normalize the distribution. Nadir CD4 and current HIVL data were not available for one and three PLWH participants, respectively. All PLWH participants were prescribed ARV medications. See Table 1 for group characteristics.

**Table 1:** Demographic, neuropsychiatric, and cognitive characteristics of the participant groups.

	HC Low-ELS (*N* = 36)		HC High-ELS (*N* = 33)		HIV Low-ELS (*N* = 26)		HIV High-ELS (*N* = 33)				
Variable	Mean	SD	Mean	SD	Mean	SD	Mean	SD	*F*/*t*/χ^2^	df	*p*
Recruitment site (% Providence, RI)	25		15		19		30		2.45	3	0.485
% New York, NY	75		85		81		70				
Age (years)	48.25^a^	11.39	48.42^b^	11.67	41.23^a,b^	9.99	45.00	10.00	2.83	3,124	**0.041**
Education (years)	14.64	2.37	15.39^a,b^	2.66	13.50^a^	2.44	14.15^b^	2.46	3.08	3,124	**0.030**
WTAR SS	104.03	14.53	106.63	13.54	102.00	12.89	102.77	17.74	0.55	3,120	0.646
% Male	58		58		65		64		0.58	3	0.901
Racial composition (% European American)	17		24		8		30		5.20	3	0.157
% African American	67		49		80		52				
% Asian American	0		0		0		3				
% Native American	0		3		0		3				
% Bi/Multiracial	5		15		8		9				
% None of the above	11		9		4		3				
Ethnic composition (% Hispanic)	25		24		12		12		3.42	3	0.332
Nadir CD4 (cells/μL)					217.92	194.06	249.15	229.78	0.55	56	0.586
Current CD4 (cells/μL)					571.38	303.87	689.58	320.66	1.44	57	0.156
Current log_10_ HIVL					2.34	1.31	1.78	0.83	1.84	36.30	0.074
% with HIVL below 50 copies/mL					50		63		0.88	1	0.350
Length of HIV infection (years)					13.15	6.46	14.76	7.54	0.86	57	0.392
% on ARV medications					100		100				
% Hepatitis C positive	6		3		8		9		1.14	3	0.769
KMSK—Alcohol (/13)	4.78^a,b^	2.88	6.03	4.04	7.73^a^	4.14	7.27^b^	3.14	4.52	3,124	**0.005**
KMSK—Cocaine (/16)	1.67^a,b^	3.63	1.97^c^	4.69	4.54^a^	6.17	7.00^b,c^	6.40	7.54	3,124	**<0.001**
KMSK—Opiate (/13)	0.72	2.09	0.30	1.13	0.77	2.60	0.48	1.50	0.42	3,124	0.737
Number of ACEs	0.83^a,b^	0.81	5.09^a,c^	2.26	1.08^c,d^	0.94	5.24^b,d^	2.21	65.20	3,124	**<0.001**
Neuropsychiatric composite (*z*-score)	−0.28^a,b^	0.80	0.30^a^	0.92	−0.15^c^	0.60^b,c^	0.43	1.09	5.06	3,124	**0.002**
1-back trial response rate (%)	98.56	3.16	98.53	2.72	97.44	3.87	97.47	4.15	1.04	3,124	0.378
1-back A′ (signal detection)	0.94	0.06	0.92	0.10	0.93	0.07	0.91	0.08	0.88	3,124	0.456
1-back mean RT latency (ms)	778.56	134.96	782.36	145.36	769.02	135.11	795.74	137.55	0.192	3,124	0.902
1-back RT-IIV (CoV)	0.26^‡^	0.06	0.24^a^	0.07	0.25^b^	0.05	0.28^a,b,‡^	0.07	3.02	3,124	**0.032**

ACE, adverse childhood events; ARV, antiretroviral; CoV, coefficient of variation, a measure of variability where higher values indicate greater variability; ELS, early-life stress; HC, HIV-negative control; HIVL, HIV viral load; KMSK, Kreek-McHugh-Schluger-Kellogg scale; RT-IIV, reaction time intra-individual variability; SS, standard score; and WTAR, Wechsler test of adult reading. Across rows, means with the same superscript are significantly different at the *p* < 0.05 level; the symbol ‡ signifies group means that differ at the trend level (*p* < 0.10).

### Early life stress quantification

Early life stress exposure was determined using a self-report measure known as the Early Life Stress Questionnaire (ELSQ; Cohen et al., 2006a). The ELSQ assesses the occurrence of 17 adverse childhood events (ACE; e.g., severe family conflict, physical abuse, emotional abuse, and neglect) prior to the age of 18 (Table 2). Participants who reported ≥3 adverse events were characterized as having high ELS and participants that reported fewer than three ACEs were characterized as low ELS, consistent with prior studies (Clark et al., 2012). Using these criteria, our HIV and HC groups were classified as follows: HC Low-ELS (*N* = 36), HC High-ELS (*N* = 33), HIV Low-ELS (*N* = 26), and HIV High-ELS (*N* = 33; Table 1).

**Table 2:** 17 adverse childhood events assessed using the Early Life Stress Questionnaire.

Adverse childhood events
Divorce
Severe family conflict
Separation from family
Surgery/hospitalization
Major illness in family
Bullied/social rejection
Death: parent/sibling
Emotional abuse
Domestic violence in home
Major illness (self)
Natural disaster
Physical abuse
Sexual abuse
War
Poverty/neglect
Fire in home
Adoption

### Reaction time task

**Figure 1:**
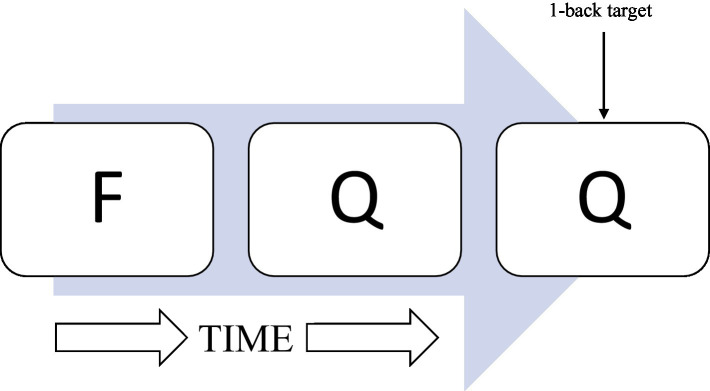
Illustration of 1-back task. A series of consonants is presented onto a computer monitor one at a time. One consonant was presented every 2 s. Participants indicated whether the letter presented was the same or different as the letter N-back, where “N” represented the specific number of letters back.

The N-back is a computerized working memory task that was used to determine RT-IIV. This task is described in detail in a prior report (Clark et al., 2018). Briefly, the task entails a series of letters presented visually, one every 2 s. Several N-back conditions were presented (e.g., 0-back, 1-back, and 2-back) during functional magnetic resonance imaging (fMRI; data not examined in the current report). Reaction time data during the 1-back condition were examined based on prior observations of ELS-related (Clark et al., 2018) and HIV-related (Chang et al., 2001) effects among PLWH. During the 1-back, participants indicate (yes/no) whether the letter presented is the same as, or different from, the letter presented immediately prior (Figure 1). As described previously (Clark et al., 2018), the N-back was completed during an magnetic resonance imaging (MRI) session as part of a broader study of ELS-related effects among PLWH. A total of 64 1-back trials were administered. Participants who demonstrated accuracy levels below chance or responded to less than 80% of 1-back trials were not included, resulting in the exclusion of 16 participants (six HC Low-ELS, four HC High-ELS, two HIV Low-ELS, and four HIV High-ELS) from the sample described herein. Those excluded did not differ significantly with respect to age (*p* = 0.991), gender (*p* = 0.544), or estimated premorbid intelligence (*p* = 0.356) when compared to the sample described herein.

We calculated a nonparametric measure of signal detection, A′ (Snodgrass and Corwin, 1988), which accounts for both omission and commission errors, and thus characterizes performance during the 1-back. A′ ranges from 0 to 1, with scores ≥0.5 indicating performance that is at or above chance. For each participant, mean RT latency was calculated using data from all correct 1-back trials. To assess RT-IIV, we calculated the coefficient of variation (CoV) for each participant using the following formula: CoV = standard deviation across all correct 1-back trials/mean RT (Stuss et al., 2003). Therefore, CoV is measure of RT variability which controls for mean RT, where higher scores indicate greater variability.

### Neuropsychiatric measures

We examined neuropsychiatric symptoms known to be both elevated among PLWH (Clark et al., 2017) and associated with RT-IIV performance (Clark et al., 2018). To examine neuropsychiatric symptoms, we created a neuropsychiatric symptom composite index score to quantify overall psychological difficulty. This index included: The Center for Epidemiological Studies-Depression Scale (CESD; Radloff, 1977), Perceived Stress Scale (PSS; Cohen et al., 1983), and Posttraumatic Stress Disorder Checklist—Civilian (PCLC; Weathers et al., 1993), which assessed current levels of depression, stress, and posttraumatic stress disorder (PTSD) symptoms, respectively. PCLC scores were not available for three PLWH participants. Consistent with prior studies (Clark et al., 2017, 2018), for each participant, *z*-scores were calculated for each measure based on the HC sample mean for that measure. Next, the three *z*-scores were averaged to generate a composite index score for each participant. Hence, the index signifies overall degree of neuropsychiatric difficulty reported across all cognitive domains assessed where higher index scores indicate greater global neuropsychiatric difficulty.

### Statistical analyses

Group differences on demographic variables and neuropsychiatric index scores were assessed using one-way ANOVAs, independent-samples *t*-tests, chi-square, and Fisher’s exact tests. Our primary goals were 2-fold. First, we examined the independent and combined effects of HIV status and high-ELS exposure on RT-IIV using a two-way ANCOVA with factors of HIV status (HIV, HC) and ELS status (High, Low). Covariates were included to control for demographic variables that differed significantly between groups and also correlated significantly (as assessed by Pearson correlations) with RT-IIV (i.e., age). Partial eta-squared (η_p_^2^) was used as an indicator of effect size, where values of 0.01, 0.06, and 0.14 indicate small, medium, and large effects, respectively (Cohen, 1988). Our second goal was to explore whether RT-IIV is sensitive to psychosocial (degree of ELS exposure) and biological (HIV-disease characteristics) risk factors known to impact brain and cognition. As such, we used linear regression to examine the relation between ELS exposure, as measured by number of ACEs reported, and RT-IIV accounting for covariates as defined above. Separate analyses were conducted for the HIV and HC groups to examine associations in each group independently. Likewise, in the HIV group, we used linear regression to examine associations between HIV-disease characteristics and RT-IIV for those disease characteristics that demonstrated significant univariate associations with RT-IIV (Pearson correlations, *p* < 0.050).

## Results

### Demographic measures

Table 1 includes demographic data for all groups, including group means and statistics. The four groups were well matched on several demographic variables including estimated premorbid intelligence and 1-back accuracy (A; *p*s > 0.050); however, groups differed significantly with respect to age, education, and lifetime drug use histories for alcohol (KMSK-A) and cocaine (KMSK-C; Table 1). We observed a significant correlation between age and RT-IIV in the HC Low-ELS group only [*r*(36) = −0.341, *p* = 0.042]. RT-IIV did not correlate significantly with education, alcohol use history, nor cocaine use history in any group (*p*s > 0.050). Accordingly, only age is included as a covariate in RT-IIV analyses. Regarding HIV-disease characteristics, the HIV Low-ELS and HIV High-ELS groups were well matched with respect to nadir and current CD4 levels, current HIVL, and length of HIV infection (*p*s > 0.050; Table 1).

### Neuropsychiatric measures

Group mean neuropsychiatric composite index scores and statistics are reported in Table 1. Results from the one-way ANOVA revealed significant differences across groups [*F*(3,124) = 5.06, *p* = 0.002], with higher symptom levels observed in both High-ELS groups relative to the Low-ELS groups (Table 1) as indicated by Fisher’s LSD post-hoc tests (all *p*s ≤ 0.050). More specifically, the HC High-ELS group reported greater neuropsychiatric symptoms than the HC Low-ELS (*p* = 0.007) and HIV Low-ELS (*p* = 0.050) groups. Similarly, the HIV High-ELS group reported greater neuropsychiatric symptoms than the HC Low-ELS (*p* = 0.001) and HIV Low-ELS (*p* = 0.013) groups. Neuropsychiatric composite scores did not correlate significantly with RT-IIV in any group (*p*s > 0.050) and thus were not included as a covariate in RT-IIV analyses.

### Reaction time measures

**Figure 2:**
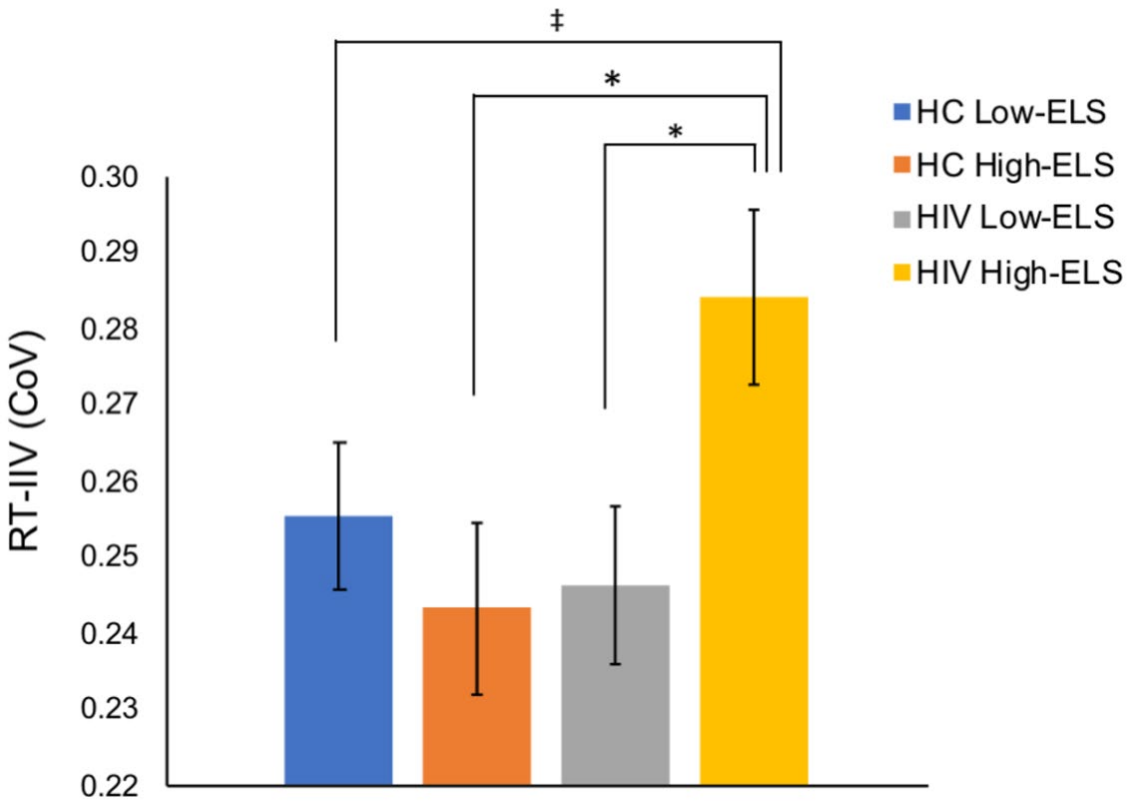
The HIV High-ELS group exhibited greater RT-IIV than all other groups. Mean (± standard error of the mean) CoV for each group are displayed. HC, HIV-negative control; ELS, early-life stress; RT-IIV, reaction time intra-individual variability; CoV, coefficient of variation, a measure of variability where higher values indicate greater variability. (^*^p < 0.05, ^‡^*p* < 0.10).

All groups exhibited a mean response rate of >97% during the 1-back (Table 1). Groups did not differ significantly in mean RT or 1-back accuracy (A′; Table 1). By contrast, results from the ANCOVA, examining RT-IIV, covarying for age (*p* < 0.001), revealed a significant HIV-ELS interaction [*F*(1,123) = 5.21, *p* = 0.024, η_p_^2^ = 0.04]. In this model, the main effects of HIV and ELS were non-significant (*p* > 0.050). Follow-up analyses revealed that the HIV-ELS interaction was driven by significant and trend-level elevations in the HIV High-ELS group relative to all other groups [HIV High-ELS versus: HC Low-ELS, *t*(67) = 1.95, *p* = 0.055; HC High-ELS, *t*(64) = 2.54, *p* = 0.013; and HIV Low-ELS, *t*(57) = 2.39, *p* = 0.020; Figure 2]. All additional group comparisons were non-significant (*p*s > 0.050).

### Relation of RT-IIV to ELS exposure

We examined the association between degree of ELS exposure and RT-IIV using linear regressions conducted in the HIV and HC groups separately. In the HIV group, we observed a significant association between ELS exposure and RT-IIV [*R*^2^ = 0.08; *F*(1,58) = 5.16, *p* = 0.027; β = 0.007, (*t* = 2.27, *p* = 0.027)]. By contrast, the association between ELS exposure and RT-IIV in the HC sample was non-significant (*p* = 0.854).

### Relation of RT-IIV to HIV-disease characteristics

Among the HIV group, RT-IIV correlated significantly with nadir CD4 [*r*(58) = −0.26, *p* = 0.049] and HIVL [*r*(56) = 0.29, *p* = 0.031] as well as with HIV-disease duration at a trend level [*r*(59) = 0.24, *p* = 0.062]. Correlations with current CD4 levels were non-significant [*r*(59) = −0.17, *p* = 0.207]. Results from the linear regression revealed a trend-level association of RT-IIV to HIVL [β = 0.014, (*t* = 1.85, *p* = 0.070)] and a non-significant association with nadir CD4 (*p* = 0.124).

## Discussion

The current study was conducted to further examine the role high-ELS exposure plays in the etiology of cognitive impairment among PLWH. In doing so, we tested the hypothesis that we would observe combined effects of HIV status and high-ELS exposure on RT-IIV—a sensitive behavioral marker of cognitive dysfunction. We report two primary findings. First, we observed that HIV High-ELS adults exhibited higher RT-IIV relative to all other groups. Consistent with this finding, combined HIV-high-ELS effects on cognitive function have been reported previously (e.g., Clark et al., 2012; Spies et al., 2012, 2016, 2017). Importantly, the current data help to further clarify the effects of ELS on RT-IIV in PLWH. Although prior data indicated high-ELS status is associated with elevated RT-IIV in adults living with HIV, comparisons to HIV-negative adults were lacking. It thus remained unclear whether increases in RT-IIV observed in HIV High-ELS adults reflected effects associated with high-ELS exposure exclusively or the combined effects of high ELS and HIV. Accordingly, our current data provide evidence of combined effects of HIV and high ELS on RT-IIV, which suggest HIV-related neural abnormalities may interact with ELS-related mechanisms in an additive or synergistic manner to increase cognitive dysfunction. Second, in line with this notion, we observed that greater degree of ELS exposure correlated with greater RT-IIV elevations in the HIV group but not in the HC group, further suggesting that HIV-related neural abnormalities may exacerbate underlying ELS-related mechanisms impacting cognitive function in a dose-related manner. Such data underscore the need to better understand whether and how ELS-related pathophysiological mechanisms may interact with those associated with HIV to increase neurocognitive dysfunction among PLWH.

Few studies report on the neural etiology of increased RT-IIV in the context of HIV and ELS, with prior data from PLWH implicating ELS-related reductions in global gray and white matter volumes (Clark et al., 2018). By contrast, numerous studies in non-HIV samples have been conducted in which the neural correlates of RT-IIV elevations are reported to include white matter volume reductions (Walhovd and Fjell, 2007), frontal lobe white matter hyperintensity burden (Bunce et al., 2007), and white matter integrity reductions (Fjell et al., 2011), as well as increased activation of inhibitory networks including frontal-lobe regions (Bellgrove et al., 2004), reduced dopaminergic neurotransmission (MacDonald et al., 2006), reduced anterior cingulate cortex (ACC) activation (Johnson et al., 2015) and volume (Albaugh et al., 2017), presence of amyloid beta pathology (Lu et al., 2020), and suppression of the default mode network (DMN; Weissman et al., 2006; Kelly et al., 2008). Several of these correlates, such as white matter volume reductions, ACC volume reductions, and DMN abnormalities are observed in association with both HIV (Clark and Cohen, 2010; Cohen et al., 2010a,b; Clark et al., 2015; Jensen et al., 2019; Hall et al., 2021; Yoshihara et al., 2022) and high ELS (Cohen et al., 2006b; Frodl et al., 2012; Philip et al., 2013; Corbo et al., 2016; Clark et al., 2018). Thus, our results, which suggest combined effects of HIV and high-ELS exposure on RT-IIV, implicate white matter abnormalities and other neural disruptions that co-occur in HIV and high-ELS samples (e.g., DMN dysfunction) in the etiology of the observed RT-IIV elevations. Yet, further research is needed to verify this hypothesis and parse out the specific pathophysiological mechanisms responsible for RT-IIV elevations and other cognitive impairments noted among PLWH who have high-ELS exposure.

Certainly, there is increasing evidence suggesting that RT-IIV could serve as a potential marker of cognitive dysfunction in HIV (Ettenhofer et al., 2010; Clark et al., 2018). Findings from the current study provide further support to this notion, as we report that RT-IIV is sensitive to risk factors known to impact cognition among PLWH, including both psychosocial (high-ELS exposure) and biological (HIVL) factors. These results are consistent with prior data indicating associations between RT-IIV elevations and measures of HIV-disease severity (e.g., current HIVL and nadir CD4 levels; Ettenhofer et al., 2010). Further, these results expand our understanding of the sensitivity of RT-IIV to psychosocial risk factors such as high-ELS exposure, particularly among PLWH. Taken as a whole, our findings support RT-IIV as a potentially useful marker of cognitive dysfunction resulting from biological or psychosocial risk factors in PLWH.

Results from the present study should be considered in the context of its limitations. While prior RT-IIV studies have utilized similar sample sizes (Ettenhofer et al., 2010) studies with larger samples may offer greater generalizability. More specifically, including larger sample sizes in future RT-IIV studies would permit investigation of factors such as age and other relevant demographic variables (e.g., substance use) that could potentially moderate and compound observed effects. An additional limitation includes the lack of a full neuropsychological battery, which future studies could examine to assess potential cognitive domain specific effects of ELS on cognitive function beyond RT-IIV.

In summary, we report novel findings reflecting the combined effects of HIV and high-ELS status on RT-IIV. We also report the additional novel observation of significant associations between degree of ELS exposure and RT-IIV among PLWH. This study adds to the growing body of literature suggesting that high ELS is a risk factor for cognitive impairment among PLWH. Additional studies are needed to understand whether and how ELS-related neural changes interact with those associated with HIV to contribute to neurocognitive dysfunction in PLWH with the goal of illuminating potential pathways and mechanisms for therapeutic intervention.

## Data availability statement

The raw data supporting the conclusions of this article will be made available by the authors, without undue reservation.

## Ethics statement

The studies involving human participants were reviewed and approved by Institutional Review Boards at the Icahn School of Medicine at Mount Sinai and The Miriam Hospital. The patients/participants provided their written informed consent to participate in this study.

## Author contributions

UC, JS, RK, RM, MP, RH, and MD contributed to design of the study, complying literature, and manuscript review. UC and RK organized the database. UC, JS, RK, and MP performed the statistical analysis. RK and JS wrote the first draft of the manuscript. All authors contributed to the article and approved the submitted version.

## Funding

This work was supported by grants from the National Institutes of Health [Grants K23 MH096628 (UC), R25 MH080663 (UC), and R25 MH083635 (UC)], and the Brown University MRI Research Facility (UC).

## Conflict of interest

The authors declare that the research was conducted in the absence of any commercial or financial relationships that could be construed as a potential conflict of interest.

## Publisher’s note

All claims expressed in this article are solely those of the authors and do not necessarily represent those of their affiliated organizations, or those of the publisher, the editors and the reviewers. Any product that may be evaluated in this article, or claim that may be made by its manufacturer, is not guaranteed or endorsed by the publisher.

## Author disclaimer

The views expressed in this article are those of the authors and do not necessarily reflect the position or policy of the NIH.
